# Drug Trafficking into Macrophages via the Endocytotic Receptor CD163

**DOI:** 10.3390/membranes5020228

**Published:** 2015-06-23

**Authors:** Jonas Heilskov Graversen, Søren Kragh Moestrup

**Affiliations:** 1 Institute of Molecular Medicine, University of Southern Denmark, J. B. Winsløws Vej 25, 5000-Odense C, Denmark; E-Mail: smoestrup@health.sdu.dk; 2 Department of Clinical Biochemistry and Pharmacology, Odense University Hospital, 5000-Odense C, Denmark

**Keywords:** macrophage, CD163, dexamethasone, antibody drug conjugate, targeting, inflammation, internalization

## Abstract

In inflammatory diseases, macrophages are a main producer of a range of cytokines regulating the inflammatory state. This also includes inflammation induced by tumor growth, which recruits so-called tumor-associated macrophages supporting tumor growth. Macrophages are therefore relevant targets for cytotoxic or phenotype-modulating drugs in the treatment of inflammatory and cancerous diseases. Such targeting of macrophages has been tried using the natural propensity of macrophages to non-specifically phagocytose circulating foreign particulate material. In addition, the specific targeting of macrophage-expressed receptors has been used in order to obtain a selective uptake in macrophages and reduce adverse effects of off-target delivery of drugs. CD163 is a highly expressed macrophage-specific endocytic receptor that has been studied for intracellular delivery of small molecule drugs to macrophages using targeted liposomes or antibody drug conjugates. This review will focus on the biology of CD163 and its potential role as a target for selective macrophage targeting compared with other macrophage targeting approaches.

## 1. Targeting Macrophages

### 1.1. Targeting Monocytes and Macrophages in Inflammation

Monocytes and macrophages have a strong regulatory effect on inflammation [1] owing to the expression of a range of cytokines, including TNF [2,3], interleukin-1 (IL-1) [3], IL-6 [4], TWEAK [5] and B-cell activation factor [6]. All of these cytokines are single targets for registered or experimental therapy with monoclonal antibodies that bind to and neutralize the effect of the cytokines. A more potent approach would be to inhibit the production of these cytokines at the source by modulating macrophage expression pattern through overall modification of macrophage polarization [7]. This may also inhibit paracrine signaling of released cytokines that might be difficult to neutralize by a therapeutic antibody due to the cell contacts. In addition, modulating macrophage phenotype may also reduce macrophage-mediated tissue damage (hemophagocytosis) in sepsis and in the macrophage activation syndrome [8,9].

### 1.2. Targeting Macrophages in Cancer

In cancer, tumor-associated macrophages are recruited to the tumor stroma where they sustain tumor-growth by (1) the removal of cell debris; (2) stimulation of neovascularization and tissue re-modeling; (3) production of growth factors; and (4) immunosuppression. Furthermore, tumor-associated macrophages contribute to metastasis by promoting tumor cell extravasation [10,11]. The tumor-supportive macrophage population is therefore an attractive target for therapies inhibiting tumor growth, in analogy with anti-angiogenic therapy that indirectly hits the tumors As an alternative to eradication with a targeted toxin, macrophages might instead be targeted with small molecule drugs to reprogram the macrophages to a repolarized state where they suppress rather than stimulate tumor growth [11].

### 1.3. Targeting Macrophages in Infectious and Lysosomal Storage Diseases

In certain infectious diseases such as HIV, tuberculosis and leishmaniasis, the macrophage serves a special function as reservoir for the pathogen. Specific targeting of macrophages with agents toxic to the infectious substance may increase the therapeutic index and enable eradication of the pathogen [12]. In leishmaniasis, the macrophage hosts the pathogen, and a liposomal formulation of the anti-leishmaniasis drug Amphotericin B is on the market for treatment of leishmaniasis. Although the liposomal formulated drug exhibits a higher therapeutic index than free drug, there are still severe adverse effects. Higher macrophage selectivity could be a way of improving the efficacy [13].

Gaucher’s disease is a lysosomal storage disease caused by lack of functional expression of the enzyme β-glucocerebrosidase in macrophage lysosomes. The disease can be treated to some extent by enzyme replacement therapy using a mannosylated version of the β-glucocerebrosidase, which owing to this carbohydrate modification is taken up through the mannose-receptor, predominantly in macrophages [14].

## 2. Using CD163 for Macrophage Targeting of Small Molecules

### 2.1. CD163 Background

CD163 was first described as a 130 kDa glucocorticoid-regulated surface protein in monocytes and macrophages [15,16]. The function of the protein was unknown until it was identified as the scavenger receptor of the complex of haptoglobin (Hp) and hemoglobin (Hb) (Hp-Hb) formed after the lysis of red blood cells [17]. Later, CD163 has been reported to bind human pathogenic bacteria [18] and tumor necrosis factor-α-like weak inducer of apoptosis (TWEAK) [19]. Furthermore, porcine CD163 has been shown to bind certain virus strains [20,21].

### 2.2. CD163 Structure

CD163 consists of nine SRCR domains, a transmembrane segment and a short C-terminal cytoplasmic tail [22]. In addition to the membrane-bound CD163, a soluble version of CD163 (sCD163) is present in plasma [23] due to cleavage of CD163 on the cell surface by the enzyme ADAM17 [24]. Three splice variants of membrane-bound CD163 resulting in three isoforms with different cytoplasmic tails have been described [22,25,26]. All three variants have the capacity to endocytose Hp-Hb, but owing to the expression level and subcellular localization, the variant with the shortest cytoplasmic tail seems responsible for the majority of the ligand clearance in man [27].

The nine SRCR domains of CD163 belongs to an ancient extracellular domain family [28], the bona fide domain consists of approximately 100 amino acids residues and it is found as a class A or B variant, which only differ by the presence of an additional disulfide bridge in the class B variant [29]. Whereas the class B SRCR domains are encoded in one exon and strictly found in vertebrates, the class A are encoded by two exons and found in all phyla from metazoa [30]. Furthermore, class A repeats are usually seen as single domains in mosaic proteins in contrast to the class B repeats, which appear as multirepeat SRCR clusters in the extracellular domains of membrane proteins [28]. Concordantly, the SRCR domains of CD163 are belonging to class B.

SRCR domains contain consensus calcium binding sites that coordinate acidic amino acids for electrostatic interactions with ligands [29], as is the case for many other extracellular domains designed for molecular interactions, like LDLR A repeats, CUB domains and others [31]. Four of the SRCR domains of CD163 (domains 2, 3, 7 and 9) have conserved consensus calcium binding sites, whereas domain 5 has a potentially/semi-conserved site. The other four SRCR of CD163 domains have at least one non-conservative mutation of essential residues in the consensus calcium binding positions [32].

### 2.3. CD163 Function

The CD163-mediated macrophage clearance of the Hp-Hb complex from circulation is a well-documented mechanism [17]. This scavenger receptor function prevents the toxic effect of the heme molecule. The Hp-Hb complex is formed after the release of hemoglobin from erythrocytes during intravascular hemolysis. Under physiological conditions, this pathway may account for approximately 10% of the red blood cell degradation, corresponding to 0.5–1 gram of hemoglobin being cleared by CD163 per day [33,34,35]. However, during excessive pathological hemolysis CD163-mediated hemoglobin removal may increase greatly. The pathway should therefore be seen as an important defense mechanism to prevent the toxic effects of heme and hemoglobin during hemolytic disease. The defense is further enforced by the CD91-mediated uptake of heme-hemopexin complexes, when heme is released during excessive hemolysis and Hp depletion [36].

The Hp-Hb complex is very tightly associated with an apparent association constant of 10^15^ M in humans [37], making it the strongest protein–protein interaction described in plasma. The crystal structure of porcine and human Hp-Hb has been determined [38]. A loop region of Hp previously identified as being involved in the interaction with CD163 [39] is localized near the Hb α-chain in the complex, and Hb binding is suggested to stabilize the loop orientation [38]. The binding to CD163 involves SRCR domain 2 and 3 of CD163 [40] and is strictly dependent on calcium [41].

After internalization in the macrophage, Hp-Hb is released from CD163 in the endosome [25] owing to the lowering of pH and calcium concentration [41], a common mechanism that ensures ligand dissociation after internalization of ligand-receptor pairs [31]. The macrophage, which is also responsible for the erythrophagocytosis of outdated red blood cells, has the enzymatic machinery for subsequent degradation of Hb in the lysosome. Heme is released and diffuses into the cytoplasm where it is degraded to biliverdin, CO and iron by heme-oxygenases, whereupon biliverdin is further degraded to bilirubin. By means of albumin, the bilirubin is then transported to the hepatocytes for conjugation and secretion into the bile [42]. Biliverdin, bilirubin and CO are reported to exert anti-inflammatory effects [43] leading to a localized anti-inflammatory response [42] partly mediated by IL-10. This in turn up-regulates both CD163 and hemeoxygenase-1 thus further potentiating Hb uptake [29,44].

During conditions of excessive intravascular hemolysis Hp can be depleted from the blood, and in this cases free Hb may also be taken up by CD163 due to a weak affinity for CD163 [45]. There seems to be important species differences on this point. In mice for instance, free Hb binds with a higher affinity and the Hp complex formation has apparent only minor effect on the affinity for murine CD163 of Hb [46]. In CD163 gene knock-out mice, Hb is cleared only slightly slower than in normal mice, albeit with a one-phase decay as opposed to a two-phase decay in wild-type mice, and Hp-Hb complex formation prior to injection does not affect pharmacokinetics in either wild-type or knock-out mice [46]. In rats, an Hp-independent mechanism of Hb clearance has also been described [47]. In conclusion, this shows crucial differences between man and rodents in Hb scavenging. In rodents, the role of Hp seems primarily to prevent renal filtration and prevent oxidative modification by Hb [48,49] rather than facilitating CD163-mediated clearance.

In addition to its function in Hb scavenging, CD163 has been reported to be acting as an erythrocyte adhesion molecule in rats [50]. Together with at least four other macrophage surface molecules, CD163 mediates adherence of erythroblasts in development to macrophages in the so-called erythroblastic island [51], thereby enhancing efficiency of late state erythropoiesis [52].

CD163 is also reported to internalize the TNF-superfamily cytokine TWEAK [19] and in this way the receptor is proposed to prevent the exertion of the pro-inflammatory signaling function of TWEAK in atherosclerosis [53]. Interestingly, soluble TWEAK and soluble CD163 seem to be inversely correlated in inflammatory cardiovascular disease, and the ratio between the two markers may increase accuracy in prediction of long-term survival in patients with peripheral arterial disease [54].

Finally, CD163 has been reported to bind pathogens such as certain vira and bacteria (*S. mutans*, *S. aureus*, and *E. coli*). Binding to CD163 on the surface of monocytic cells induces pro-inflammatory signaling, as evidenced by an increase in TNF release *in vitro* [18]. Based on this finding, CD163 has been suggested to have an innate immune related bacterial sensing function mediating a local immune response [18]. In addition, it has been shown that *S. aureus* induces shedding of CD163 from the monocyte, with sCD163 subsequently being able to bind to the bacteria trough fibronectin bound on the bacterial surface. The surface binding of fibronectin is an integral part of the pathomechanism of *S. aureus*, and CD163 binding might thereby protect against *S. aureus* infection by enhancing phagocytosis of the bacteria [55].

In addition to binding some bacteria, CD163 has been reported to bind virus particles in pigs. Contrary to the perceived role of human CD163 in defense against the pathogens, CD163 seems to promote the cellular entry of the virus. This is the case in the infection with the African swine fever virus (ASFV) and porcine reproductive and respiratory syndrome virus (PRRSV) [56]. For ASFV, the role of CD163 is to act as a point of attachment for subsequent macrophage internalization leading to infection of the monocyte/macrophage, an interaction that can be inhibited by a specific CD163 monoclonal antibody [20]. For PRRSV, the role of CD163 seems related to virus un-coating in the early endosome following sialoadhesin-mediated internalization, thus rendering the virus infective [21,57]. A central region of CD163, mainly consisting of SRCR domain number 5, which is different from the Hp-Hb binding region, is involved in the interaction with the virus [58].

Interestingly, human monocytes/macrophages have been reported to be more permissive to HIV infection *in vitro* after substance P-mediated increase of CD163 expression, and HIV infection of monocytes can be inhibited by Hp-Hb. However, the data needs further conformation [59].

### 2.4. CD163 Expression

CD163 is expressed only in cells of the monocytic-macrophage lineage, and with increasing expression as monocytes maturate into macrophages. The expression of CD163 is especially high in macrophages in liver (Kupffer cells), red pulp of the spleen, the lung and the bone marrow [60]. Other resident monocyte-derived cells such as Langerhans [61] and dendritic cells [62] do not, or only weakly, express CD163.

Varying levels of CD163 expression on monocytes and macrophages have been reported in literature and this confusing discrepancy is due to different features of the antibodies used in the different studies. For instance, binding of some antibodies is sensitive to EDTA used as anti-coagulant; others recognize CD163 epitopes that are less accessible when the receptor is inserted in the membrane [32].

Culturing of monocytes *in vitro* greatly increases the expression level of CD163 [16]. Further, CD163 expression level can be up-regulated by stimulation with a range of reagents affecting the maturation of monocytes into specific macrophage subtypes. *In vitro*, CD163 expression on monocytes (and maturated macrophages) can be upregulated by heme, Hb, glucocorticoids, IL-6, and IL-10, whereas lipopolysaccharide (LPS), TNF, IL-4, and granulocyte-macrophage colony stimulating factor down-regulate expression [63].

For many years, CD163 has been seen as a classical marker for human M2-macrophages, also designated “alternatively activated” macrophages, albeit this is an inherent *in vitro* distinction [64] because *in vivo* the macrophage has a rather plastic phenotype that responds to the many local and different stimulations. CD163 expression on M2-like macrophages has been shown in a range of inflamed tissues in both chronic and acute inflammation [63]. Table 1 displays a list of inflammatory diseases where CD163 expressing macrophages have been identified at the site of inflammation, and for which cytokine signaling is part of the disease pathomechanism. In addition, high levels of sCD163 can be detected in plasma in a wide range of inflammatory diseases and most likely reflects a general increase of CD163 expression at sites of inflammation [65]. Increased levels of CD163 expressing macrophages are also found in the microglia of Alzheimer’s disease patients’ frontal and occipital cortices and in the brainstems of Parkinson’s disease patients, the CD163 expressing cells could either be resident microglial macrophages or infiltration of the brain by systemic macrophages [66]. In HIV patients with neurocognitive impairment, the macrophages at the sites of neuroinflammation are activated and exhibit increased CD163 expression [67]. In rhesus macaque monkeys, it has been shown that the activation and increase in CD163 expression is caused by macrophage colony-stimulating factor [68]. Further, in SIV infected monkeys, increased numbers of CD163+ macrophages in the heart is correlated with increased cardiac fibrosis and myocardial degeneration [69].

**Table 1:** Inflammatory indications with up-regulation of CD163(+) macrophages at site of inflammation, as evidenced by studies of patient samples.

Inflammatory Conditions with Up-Regulation of CD163 Macrophages at Site of Inflammation and Involvement of Pro-inflammatory Cytokines in Pathogenesis			
Acute and infectious inflammations		Chronic sterile inflammations	
Indication	Reference	Indication	Reference
Acute alcoholic hepatitis	[70,71]	Non-alcoholic steatohepatitis (NASH)	[72,73]
Acute viral hepatitis	[74]	Rheumatoid arthritis	[75,76,77,78]
Sepsis	[79]	Psoriatic arthritis	[80]
Hemophagocytic syndrome	[81,82]	Giant cell arteritis	[83]
Celiac disease	[84]	Osteoarthritis	[85,86]
Acute kidney failure	[87,88]	Graft *versus* host disease	[89]
Rejected kidney allografts	[90]	Inflammatory bowel disease	[91,92,93]
Atherosclerosis	[53,94,95]	Multiple sclerosis	[96,97]
HIV	[67]	Sarcoidosis	[98,99]
		Scleroderma	[100,101,102]
		Chronic obstructive pulmonary disease	[103]
		Systemic lupus erythematosus	[104,105]
		Alzheimer’s disease	[66]

Tumor-associated macrophages also exhibit a prominent CD163 expression the level of CD163 expression is linked to poor survival in a range of tumors, as listed in Table 2.

**Table 2:** Cancer indications with proven negative correlation between tumor-associated macrophage CD163 expression and survival.

Cancers with Link between Tumor-Associated Macrophage CD163 Expression and Survival	
Cancer tissue	Reference
Myeloma	[106]
T-cell lymphomas	[107,108]
Hodgkin’s lymphoma	[109,110,111]
Follicular lymphoma	[112]
Meningioma	[113,114]
Glioma	[115]
Epithelial ovarian cancer	[114,116]
Non-small cell lung cancer	[117]
Pancreatic cancer	[118]
Stroma of breast cancer	[119,120]
Stroma of bladder cancer	[121]
Oral squamous cell cancer	[122,123]
Colorectal cancer	[124]
Papillary renal cell carcinoma	[125]
Clear cell renal cell carcinoma	[126]
Endometrial adenocarcinoma	[127]
Intrahepatic cholangiocarcinoma	[128]
Uveal melanoma	[129]
Cutaneous malignant melanoma	[130,131]

In addition to expression on monocyte-derived cells, expression of CD163 and other macrophages antigens on cancer cells have been described [132]. It has therefore been hypothesized that tumor cells can fuse with macrophages, and this has now been documented in *in vitro* studies [133,134,135,136,137]. Such fusion cells grow slower *in vitro* [138], whereas *in vivo* they grow faster and exhibit higher metastatic potential compared to normal cancer cells [136,139,140]. CD163 expression on the cancer cells has been demonstrated in 48% of breast cancer biopsies [141], 39% of bladder cancer biopsies [135], 23% of rectal cancer biopsies [142] and 20% of colorectal cancer biopsies [124]. In all the studies expression, the level of CD163 on the cancer cells correlated with poor survival [143].

Finally, CD163 expression has been described on primary tumors of monocytic origin, such as histiocytic sarcomas [144], and certain subtypes (M4/M5) of acute myeloid leukemia [145,146].

### 2.5. CD163 as a Target for Rapid Internalization of Vehicles

CD163 seems to be an ideal target for intracellular delivery of drugs to macrophages, because of its highly selective expression in the tissue of interest and its function as a constitutive endocytotic receptor [147,148]. The rapid internalization of ligands binding to CD163 ensures minimum systemic exposure of the drug. The rapid clearance is evidenced by injections of monoclonal antibodies in animals. In pigs, a humanized CD163 monoclonal antibody injected at a dose of 2 mg/kg exhibits a plasma half-life in the range of 5–8 min [149]. For a CD163 monoclonal antibody injected at a dose of 2.4 mg/kg in rats, 50% of the dose is cleared after 20 min [150]. An i.v.-injected ^68^Ga-labeled CD163 antibody showed fast clearance and rapid accumulation to macrophage-rich tissues by PET-scanning. The majority of the dose was taken up by the liver, whereas the spleen exhibited the highest dose relative to organ weight [151]. In rat liver *in vivo*, the ^68^Ga-labeled CD163 antibody was specifically binding to and internalized in Kupffer cells [151]. Furthermore, in arthritic rats there was an increased deposition of antibodies in the inflamed paws of the animals compared to healthy rats.

So-called stealth liposomes with reduced uptake in phagocytosing cells can be created by PEGylation of the liposome surface [152] with polyethylene glycol (PEG). Using a PEG molecule as linker, the surface of the liposome can be further modified, for instance with monoclonal antibodies (mAbs) ensuring liposome binding to specific cell type antigens [153]. PEGylated stealth liposomes linked with CD163 antibodies on the surface have also shown rapid clearance in rats and accumulation in macrophage-rich tissues [154]. Liposomes with human Hb as the targeting moiety instead of a CD163 antibody also exhibited specific uptake in CD163-expressing macrophages [155]. Targeted liposomes enables incorporation of a wider range of drugs than can be achieved using antibody drug conjugates (ADCs) and further, more drugs can be delivered per cell per unit taken up compared to ADCs.

The elevated plasma concentration of sCD163 in patients with inflammation might theoretically compete for ADC binding to CD163. However, the sCD163 in plasma is low compared to the expression level of membrane-bound CD163, which has the further advantage of increasing avidity by being cross-linked by bi- or multivalent ligands. Moreover, in a therapeutic setting the therapeutic dose of antibodies may neutralize the sCD163 after the first injection of ADC.

In accordance with an endocytic uptake of antibody-drug complexes via CD163, cellular uptake studies have shown that antibody and drug co-localize in intracellular compartments corresponding to endosomes or lysosomes after 30 min. These studies with dexamethasone-conjugated antibody against rat CD163 have taken advantage of an esterase degradable linker enabling intracellular release of dexamethasone in the endosome/lysosome. The segregation of antibody and drug was visible after 2 h [150]. Similar uptake kinetics has been observed with CD163-directed liposomes [154].

A drug delivered to the macrophage endosome/lysosome can then be released and, given that it has the right properties in terms of membrane permeability, diffuse passively into the cytosol where it exerts its function. The clearance of the CD163 binding vehicle, due to clearance and uptake in macrophages, is fast, so it is preferable to have a prolonged (e.g., drugs regulating translation/transcription such as glucocorticoids [156,157] or siRNA [158]) or an irreversible drug effect (e.g., cytotoxins) on the cell. A CD163-targeting drug will be systemically distributed to monocytes and macrophages of perfused organs. However, a targeting vehicle will probably not be able to cross the blood–brain barrier and reach the CD163-expressing microglia. Nevertheless, the drugs could anyway have an effect by targeting circulating monocytes prior to their migration over the blood–brain barrier.

### 2.6. Effect of Targeting Macrophages with Small Molecule Drugs Using CD163

The CD163-targeting of glucocorticoid has a tremendous effect as shown *in vitro* and *in vivo* [149,150,159]. The *in vivo* anti-inflammatory effect of anti-CD163 IgG-dexamethasone in rats [150] is comparable to a 50-fold higher dose of free dexamethasone in terms of inhibiting LPS-induced cytokine production. This study also shows that by the targeting to CD163 with low dose dexamethasone, serious side effects (e.g., reduced body weight, lymphocytes apoptosis and suppression of endogenous cortisol production) of the equipotent high doses of free dexamethasone are avoided [150]. The anti-inflammatory equipotent 50-fold lower dose of dexamethasone (0.02 mg/kg) as a CD163 targeting ADC does not affect any of these systemic parameters compared to vehicle treatment [150].

The findings in rats have been corroborated by a similar study in a pig endotoxemia model [149]. In this study the anti-CD163-dexamethasone ADC was based on a humanized CD163 mAb linked with dexamethasone trough the same esterase-sensitive linker as used in rats [149]. The effect on cytokine production was again comparable to a 50-fold higher dose of free dexamethasone (0.02 *vs.* 1 mg/kg), and again adverse systemic effects of the ADC was not observed, as evidenced by unchanged cortisol and ACTH levels compared to vehicle treatment.

In addition to modulating macrophage phenotype, direct eradication of macrophages may also be of interest, for instance in reducing tumor growth by removing tumor-associated macrophages. Eradication of macrophages including tumor-associated macrophages using clodronate-liposomes has accordingly been shown to slow the growth of a chemically induced lung tumor and a cutaneous T-cell lymphoma in mice [160,161] as well as to inhibit tumor angiogenesis in murine cancer models [162,163]. Further, in these mice studies macrophage depletion for up to five weeks did not reveal serious acute adverse effects. Macrophage depletion using clodronate liposomes has also been attempted in dogs for treatment of hemolytic anemia [164], soft tissue sarcoma [165] and malignant histiocytosis [166]. In these studies, temporary macrophage depletion was induced for 2–8 weeks, and no serious adverse effects were observed. However, in pigs, depletion of aveolar macrophages rendered the pig more sensitive to human H1N1 influenza virus, causing a mortality rate of 40% [167]. Similarly, impaired aveolar macrophage function has been linked to increased mortality to primary respiratory syncytial virus bronchiolitis, as evidenced by studies of human lung tissue and confirmed by murine studies [168]. This clearly points to potential adverse effects of macrophage depletion. Clodronate liposomes are not only killing macrophages but also dendritic cells [168]. Specific targeting of CD163 expressing cells should prevent depletion of dendritic cells, thus potentially further limit the adverse effects of the treatment. Linking a CD163-antibody to Doxil^TM^, a registered liposomal doxorubicin formulation against Kaposi’s sarcoma, has been shown to specifically kill CD163-expressing cells [154]. Further studies using this approach in animal cancer models are in progress and may further validate specific macrophage-targeting as a viable approach for cancer treatment.

## 3. Other Attempts at Targeting Macrophages in Inflammation and Cancer

The targeting of macrophages with a vehicle can be also achieved by other active targeting means [12,169].

By conjugating a 29 amino acid peptide derived from rabies virus glycoprotein to TNF-silencing siRNA, active targeting and uptake in macrophages and dendritic cells have been demonstrated [170]. The construct lowered LPS-induced TNF production by macrophages and dendritic cells both *in vitro* and *in vivo* [171].

Macrophage targeting approaches also include the linking of drugs with specific carbohydrates that recognize lectin receptors on the macrophage surface. One attempt has been targeting of TNF-silencing siRNA by linking it to *β*-1,3-glucan schizophyllan that binds to dectin-1 on the surface on antigen presenting cells. This accomplished binding to and internalization in antigen-presenting cells, including macrophages [172]. The construct was taken up in the Kupffer cells of the liver, and it protected mice from hepatitis induced by the combination of LPS and D-galactosamine [172].

Another approach using carbohydrates has been to target the mannose receptor. As mentioned earlier in this review, this approach is used for targeting β-glucocerebrosidase for treatment of the lysosomal storage disease Gaucher’s disease. By conjugating dexamethasone to mannosylated human serum albumin, uptake in Kupffer cells has been demonstrated. However, in this study, there was also an increase in unexplained liver fibrosis. The effect seemed to be related to the mannosylation of serum albumin, because mannosylated serum albumin without dexamethasone also caused increased liver fibrosis [173].

Mannosylated liposomes have also been tried and, when loaded with dexamethasone and administered intratracheally in rats, they were about five-fold more effective than free dexamethasone [174]. At a dose of 0.5 mg/kg, the liposomes inhibited cytokine signaling and lung damage in LPS-induced lung inflammation in rats [175]. Though encouraging, the dexamethasone dose used is still quite high, and might, even if administered systemically, still cause severe adverse effects. Further, the mannose receptor is not expressed selectively on macrophages, but also on dendritic cells and a range of non-vascular endothelia including hepatic, splenic, lymphatic, endothelia, as well as on kidney cells, mesangial cells, and trachea smooth muscle cells [176], leading to potential off-target uptake in other cell types. In this perspective, the mannose receptor seems less favorable for specific delivery to macrophages.

As an alternative to active targeting, macrophages can be hit by passive targeting as shown in other studies targeting with liposomal glucocorticoids. Passive targeting using liposomes without targeting moiety is hampered by not being macrophage-specific, regardless of whether stealth liposomes or naked liposomes are used, with up-take in other cell types such as hepatocytes, endothelial and epithelial cells, neutrophils, dendritic cells, B-cells, M-cells and subtypes of T-cells [169]. Several studies of the effect of glucocorticoids in liposomes on animal models of inflammation have been published [177]. A study in mice with autoimmune-induced arthritis (AIA) revealed that in the arthritic joints, liposomes were primarily taken up by macrophages and by early osteoclasts precursors. This was reflected by reduced arthritis and bone erosion when mice were treated with liposomes with prednisolone [178].

Whether liposomes are used in active or passive targeting, the drug cargo should preferably be located inside the liposome and not in the liposomal membrane in order to increase the stability of the liposomes and minimize drug leakage. Further, this allows the number of drug molecules per liposome to be increased allowing a reduction in the number of liposomes injected, and thereby a reduction of the risk of adverse effects to the liposome injection *per se*. This was achieved using the so-called “remote loading technique” to encapsulate prednisolone hemisuccinate and betamethasone hemisuccinate in small (80 nm) PEGylated liposomes [179,180]. The liposomes exhibited increased plasma retention time and an increased effect in AIA rats compared to free glucocorticoid both in early and late development of disease [180]. Further, the localization of the liposomes in the paws was approximately doubled in AIA rats compared to control animals [180]. These liposomes have shown similar effect when being administrated subcutaneously, where only approximately 40% of the injected dose reached circulation [181]. In a murine experimental autoimmune encephalomyelitis model of multiple sclerosis, this liposomal glucocorticoid was more efficient than a five times higher dose of non-liposomal glucocorticoid [179].

Stealth liposomes loaded with dexamethasone have been tested in models of acute and chronic chemically induced liver injury in mice, and showed that 1 mg/kg of liposome-formulated dexamethasone inhibited liver fibrosis compared to both vehicle and free dexamethasone. However, the effect appeared mainly to be owing to T-cell depletion after uptake of the liposomes, and only to a limited extent to a re-polarization effect on macrophages [182].

## 4. Concluding Remarks

Macrophage cytokine production is of importance in the propagation of a range of inflammatory indications. The production of these cytokines has been modified through active and passive targeting of macrophages. A straightforward way is to specifically target glucocorticoids to macrophages to re-polarize the macrophage to an anti-inflammatory phenotype. Glucocorticoids are very potent and efficacious anti-inflammatory drugs. However, they also exert strong systemic adverse effects since they can modulate transcription of multiple genes in virtually all cells of the body.

This requires a more strict selectivity of the cellular targeting to reduce the adverse effects of glucocorticoids, compared to for instance targeting of siRNA, which only directly affects a single gene, which may only be expressed in a limited range of cells. Using both passive and active targeting of dexamethasone to macrophages increases in efficacy have been achieved, but the dose used was still relatively high and able to cause systemic adverse effects. This was also the case when the mannose receptor was used as target, even though it is expressed at a high level on macrophages, since the macrophage expression specificity is relatively limited. More macrophage-specific targets should thus be used in order to obtain an even larger reduction in the dose of glucocorticoid needed to exert an anti-inflammatory effect.

Specific macrophage targeting can be obtained by targeting the macrophage receptor CD163. Targeting dexamethasone to macrophages using an anti-CD163-dexamethasone ADC increased the anti-inflammatory effect of dexamethasone in terms of inhibition of cytokine production in primitive inflammation models by approximately 50-fold.

Alteration of macrophage activity using CD163-specific targeting with anti-inflammatory drugs could in principle be relevant in a range of inflammatory indications as listed in Table 1. In order to further increase the effect of targeting CD163-macrophages, initial studies could be conducted in indications affecting assessable organs with a high number of macrophages [183,184].

Specific targeting of macrophages with dexamethasone may not entirely wipe out side effects. It is reasonable to assume that adverse effects must fall within a subset of the adverse effects already observed for systemic treatment with glucocorticoids. Most notably, a change in macrophage activation may increase risk of infection, although the B- and T-cell response should still function. Activation of latent tuberculosis infection could thus be a risk, as is the case for treatment with biological TNF-inhibitors [185]. However, more is still to be learned about the long-term effect on changing and reducing macrophage activity.

In conclusion, CD163 is a promising target for specific delivery of small drugs into the macrophage, and it has been validated as a target in animal models. 

## References

[1] Szabo G., Petrasek J., Bala S. (2012). Innate immunity and alcoholic liver disease. Dig. Dis..

[2] Schett G. (2008). Review: Immune cells and mediators of inflammatory arthritis. Autoimmunity.

[3] Dayer J.M. (2004). The process of identifying and understanding cytokines: From basic studies to treating rheumatic diseases. Best Pract. Res. Clin. Rheumatol..

[4] Yao X., Huang J., Zhong H., Shen N., Faggioni R., Fung M., Yao Y. (2014). Targeting interleukin-6 in inflammatory autoimmune diseases and cancers. Pharmacol. Ther..

[5] Nazeri A., Heydarpour P., Sadaghiani S., Sahraian M.A., Burkly L.C., Bar-Or A. (2014). A further TWEAK to multiple sclerosis pathophysiology. Mol. Neurobiol..

[6] Shivakumar L., Ansell S. (2006). Targeting B-lymphocyte stimulator/B-cell activating factor and a proliferation-inducing ligand in hematologic malignancies. Clin. Lymphoma Myeloma.

[7] Martinez F.O., Gordon S. (2015). The evolution of our understanding of macrophages and translation of findings toward the clinic. Expert Rev. Clin. Immunol..

[8] Schaer D.J., Schaer C.A., Schoedon G., Imhof A., Kurrer M.O. (2006). Hemophagocytic macrophages constitute a major compartment of heme oxygenase expression in sepsis. Eur. J. Haematol..

[9] Weaver L.K., Behrens E.M. (2014). Hyperinflammation, rather than hemophagocytosis, is the common link between macrophage activation syndrome and hemophagocytic lymphohistiocytosis. Curr. Opin. Rheumatol..

[10] Noy R., Pollard J.W. (2014). Tumor-associated macrophages: From mechanisms to therapy. Immunity.

[11] Ostuni R., Kratochvill F., Murray P.J., Natoli G. (2015). Macrophages and cancer: From mechanisms to therapeutic implications. Trends Immunol..

[12] Jain N.K., Mishra V., Mehra N.K. (2013). Targeted drug delivery to macrophages. Expert Opin. Drug Deliv..

[13] Gupta S., Pal A., Vyas S.P. (2010). Drug delivery strategies for therapy of visceral leishmaniasis. Expert Opin. Drug Deliv..

[14] Friedman B., Vaddi K., Preston C., Mahon E., Cataldo J.R., McPherson J.M. (1999). A comparison of the pharmacological properties of carbohydrate remodeled recombinant and placental-derived beta-glucocerebrosidase: Implications for clinical efficacy in treatment of Gaucher disease. Blood.

[15] Pulford K., Micklem K., McCarthy S., Cordell J., Jones M., Mason D.Y. (1992). A monocyte/macrophage antigen recognized by the four antibodies GHI/61, Ber-MAC3, Ki-M8 and SM4. Immunology.

[16] Zwadlo G., Voegeli R., Schulze Osthoff K., Sorg C. (1987). A monoclonal antibody to a novel differentiation antigen on human macrophages associated with the down-regulatory phase of the inflammatory process. Exp. Cell Biol..

[17] Kristiansen M., Graversen J.H., Jacobsen C., Sonne O., Hoffman H.J., Law S.K., Moestrup S.K. (2001). Identification of the haemoglobin scavenger receptor. Nature.

[18] Fabriek B.O., van Bruggen R., Deng D.M., Ligtenberg A.J., Nazmi K., Schornagel K., Vloet R.P., Dijkstra C.D., van den Berg T.K. (2009). The macrophage scavenger receptor CD163 functions as an innate immune sensor for bacteria. Blood.

[19] Bover L.C., Cardo-Vila M., Kuniyasu A., Sun J., Rangel R., Takeya M., Aggarwal B.B., Arap W., Pasqualini R. (2007). A previously unrecognized protein-protein interaction between TWEAK and CD163: Potential biological implications. J. Immunol..

[20] Sanchez-Torres C., Gomez-Puertas P., Gomez-del-Moral M., Alonso F., Escribano J.M., Ezquerra A., Dominguez J. (2003). Expression of porcine CD163 on monocytes/macrophages correlates with permissiveness to African swine fever infection. Arch. Virol..

[21] Van Gorp H., van Breedam W., Delputte P.L., Nauwynck H.J. (2008). Sialoadhesin and CD163 join forces during entry of the porcine reproductive and respiratory syndrome virus. J. Gen. Virol..

[22] Law S.K., Micklem K.J., Shaw J.M., Zhang X.P., Dong Y., Willis A.C., Mason D.Y. (1993). A new macrophage differentiation antigen which is a member of the scavenger receptor superfamily. Eur. J. Immunol..

[23] Moller H.J., Peterslund N.A., Graversen J.H., Moestrup S.K. (2002). Identification of the hemoglobin scavenger receptor/CD163 as a natural soluble protein in plasma. Blood.

[24] Etzerodt A., Maniecki M.B., Moller K., Moller H.J., Moestrup S.K. (2010). Tumor necrosis factor alpha-converting enzyme (TACE/ADAM17) mediates ectodomain shedding of the scavenger receptor CD163. J. Leukoc. Biol..

[25] Schaer C.A., Schoedon G., Imhof A., Kurrer M.O., Schaer D.J. (2006). Constitutive endocytosis of CD163 mediates hemoglobin-heme uptake and determines the noninflammatory and protective transcriptional response of macrophages to hemoglobin. Circ. Res..

[26] Ritter M., Buechler C., Langmann T., Schmitz G. (1999). Genomic organization and chromosomal localization of the human CD163 (M130) gene: A member of the scavenger receptor cysteine-rich superfamily. Biochem. Biophys. Res. Commun..

[27] Nielsen M.J., Madsen M., Moller H.J., Moestrup S.K. (2006). The macrophage scavenger receptor CD163: Endocytic properties of cytoplasmic tail variants. J. Leukoc. Biol..

[28] Martinez V.G., Moestrup S.K., Holmskov U., Mollenhauer J., Lozano F. (2011). The conserved scavenger receptor cysteine-rich superfamily in therapy and diagnosis. Pharmacol. Rev..

[29] Graversen J.H., Madsen M., Moestrup S.K. (2002). CD163: A signal receptor scavenging haptoglobin-hemoglobin complexes from plasma. Int. J. Biochem. Cell Biol..

[30] Resnick D., Pearson A., Krieger M. (1994). The SRCR superfamily: A family reminiscent of the Ig superfamily. Trends Biochem. Sci..

[31] Andersen C.B., Moestrup S.K. (2014). How calcium makes endocytic receptors attractive. Trends Biochem. Sci..

[32] Maniecki M.B., Etzerodt A., Moestrup S.K., Moller H.J., Graversen J.H. (2011). Comparative assessment of the recognition of domain-specific CD163 monoclonal antibodies in human monocytes explains wide discrepancy in reported levels of cellular surface CD163 expression. Immunobiology.

[33] Garby L., Noyes W.D. (1959). Studies on hemoglobin metabolism. I. The kinetic properties of the plasma hemoglobin pool in normal man. J. Clin. Investig..

[34] Garby L., Noyes W.D. (1959). Studies on hemoglobin metabolism. II. Pathways of hemoglobin iron metabolism in normal man. J. Clin. Investig..

[35] Thomsen J.H., Etzerodt A., Svendsen P., Moestrup S.K. (2013). The haptoglobin-CD163-heme oxygenase-1 pathway for hemoglobin scavenging. Oxidative Med. Cell. Longev..

[36] Hvidberg V., Maniecki M.B., Jacobsen C., Hojrup P., Moller H.J., Moestrup S.K. (2005). Identification of the receptor scavenging hemopexin-heme complexes. Blood.

[37] Hwang P.K., Greer J. (1980). Interaction between hemoglobin subunits in the hemoglobin. haptoglobin complex. J. Biol. Chem..

[38] Andersen C.B.F., Torvund-Jensen M., Nielsen M.J., de Oliveira C.L.P., Hersleth H.-P., Andersen N.H., Pedersen J.S., Andersen G.R., Moestrup S.K. (2012). Structure of the haptoglobin-haemoglobin complex. Nature.

[39] Nielsen M.J., Petersen S.V., Jacobsen C., Thirup S., Enghild J.J., Graversen J.H., Moestrup S.K. (2007). A unique loop extension in the serine protease domain of haptoglobin is essential for CD163 recognition of the haptoglobin-hemoglobin complex. J. Biol. Chem..

[40] Madsen M., Moller H.J., Nielsen M.J., Jacobsen C., Graversen J.H., van den Berg T., Moestrup S.K. (2004). Molecular characterization of the haptoglobin.hemoglobin receptor CD163. Ligand binding properties of the scavenger receptor cysteine-rich domain region. J. Biol. Chem..

[41] Nielsen M.J., Andersen C.B., Moestrup S.K. (2013). CD163 binding to haptoglobin-hemoglobin complexes involves a dual-point electrostatic receptor-ligand pairing. J. Biol. Chem..

[42] Rother R.P., Bell L., Hillmen P., Gladwin M.T. (2005). The clinical sequelae of intravascular hemolysis and extracellular plasma hemoglobin: A novel mechanism of human disease. JAMA.

[43] Grochot-Przeczek A., Dulak J., Jozkowicz A. (2012). Haem oxygenase-1: Non-canonical roles in physiology and pathology. Clin. Sci. (Lond.).

[44] Philippidis P., Mason J.C., Evans B.J., Nadra I., Taylor K.M., Haskard D.O., Landis R.C. (2004). Hemoglobin scavenger receptor CD163 mediates interleukin-10 release and heme oxygenase-1 synthesis: Antiinflammatory monocyte-macrophage responses *in vitro*, in resolving skin blisters *in vivo*, and after cardiopulmonary bypass surgery. Circ. Res..

[45] Schaer D.J., Schaer C.A., Buehler P.W., Boykins R.A., Schoedon G., Alayash A.I., Schaffner A. (2006). CD163 is the macrophage scavenger receptor for native and chemically modified hemoglobins in the absence of haptoglobin. Blood.

[46] Etzerodt A., Kjolby M., Nielsen M.J., Maniecki M., Svendsen P., Moestrup S.K. (2013). Plasma clearance of hemoglobin and haptoglobin in mice and effect of CD163 gene targeting disruption. Antioxid. Redox Signal..

[47] Weinstein M.B., Segal H.L. (1984). Uptake of free hemoglobin by rat liver parenchymal cells. Biochem. Biophys. Res. Commun..

[48] Boretti F.S., Buehler P.W., D’Agnillo F., Kluge K., Glaus T., Butt O.I., Jia Y., Goede J., Pereira C.P., Maggiorini M. (2009). Sequestration of extracellular hemoglobin within a haptoglobin complex decreases its hypertensive and oxidative effects in dogs and guinea pigs. J. Clin. Investig..

[49] Buehler P.W., Abraham B., Vallelian F., Linnemayr C., Pereira C.P., Cipollo J.F., Jia Y., Mikolajczyk M., Boretti F.S., Schoedon G. (2009). Haptoglobin preserves the CD163 hemoglobin scavenger pathway by shielding hemoglobin from peroxidative modification. Blood.

[50] Fabriek B.O., Polfliet M.M., Vloet R.P., van der Schors R.C., Ligtenberg A.J., Weaver L.K., Geest C., Matsuno K., Moestrup S.K., Dijkstra C.D. (2007). The macrophage CD163 surface glycoprotein is an erythroblast adhesion receptor. Blood.

[51] Koury M.J. (2007). Scavenger receptor helps erythroblasts stay on island. Blood.

[52] Chasis J.A. (2006). Erythroblastic islands: Specialized microenvironmental niches for erythropoiesis. Curr. Opin. Hematol..

[53] Moreno J.A., Munoz-Garcia B., Martin-Ventura J.L., Madrigal-Matute J., Orbe J., Paramo J.A., Ortega L., Egido J., Blanco-Colio L.M. (2009). The CD163-expressing macrophages recognize and internalize TWEAK: Potential consequences in atherosclerosis. Atherosclerosis.

[54] Urbonaviciene G., Martin-Ventura J.L., Lindholt J.S., Urbonavicius S., Moreno J.A., Egido J., Blanco-Colio L.M. (2011). Impact of soluble TWEAK and CD163/TWEAK ratio on long-term cardiovascular mortality in patients with peripheral arterial disease. Atherosclerosis.

[55] Kneidl J., Loffler B., Erat M.C., Kalinka J., Peters G., Roth J., Barczyk K. (2012). Soluble CD163 promotes recognition, phagocytosis and killing of Staphylococcus aureus via binding of specific fibronectin peptides. Cell. Microbiol..

[56] Van Gorp H., Delputte P.L., Nauwynck H.J. (2010). Scavenger receptor CD163, a Jack-of-all-trades and potential target for cell-directed therapy. Mol. Immunol..

[57] Van Gorp H., van Breedam W., Delputte P.L., Nauwynck H.J. (2009). The porcine reproductive and respiratory syndrome virus requires trafficking through CD163-positive early endosomes, but not late endosomes, for productive infection. Arch. Virol..

[58] Van Gorp H., van Breedam W., van Doorsselaere J., Delputte P.L., Nauwynck H.J. (2010). Identification of the CD163 protein domains involved in infection of the porcine reproductive and respiratory syndrome virus. J. Virol..

[59] Tuluc F., Meshki J., Spitsin S., Douglas S.D. (2014). HIV infection of macrophages is enhanced in the presence of increased expression of CD163 induced by substance P. J. Leukoc. Biol..

[60] Pulford K., Micklem K., Law S., Mason D., Kishimoto T., Kikutani H., von dem Borne A.E.G.K., Goyert A.M., Miyasak M., Moretta L., Okumura K., Shaw S., Springer T.A., Sugamura K. (1998). CD163 (M130 antigen) workshop panel report. Leukocyte Typing VI: White Cell Differentiation Antigens.

[61] Kodelja V., Goerdt S. (1994). Dissection of macrophage differentiation pathways in cutaneous macrophage disorders and *in vitro*. Exp. Dermatol..

[62] Maniecki M.B., Moller H.J., Moestrup S.K., Moller B.K. (2006). CD163 positive subsets of blood dendritic cells: The scavenging macrophage receptors CD163 and CD91 are coexpressed on human dendritic cells and monocytes. Immunobiology.

[63] Etzerodt A., Moestrup S.K. (2013). CD163 and inflammation: Biological, diagnostic, and therapeutic aspects. Antioxid. Redox Signal..

[64] Vogel D.Y., Glim J.E., Stavenuiter A.W., Breur M., Heijnen P., Amor S., Dijkstra C.D., Beelen R.H. (2014). Human macrophage polarization *in vitro*: Maturation and activation methods compared. Immunobiology.

[65] Moller H.J. (2012). Soluble CD163. Scand. J. Clin. Lab. Investig..

[66] Pey P., Pearce R.K., Kalaitzakis M.E., Griffin W.S., Gentleman S.M. (2014). Phenotypic profile of alternative activation marker CD163 is different in Alzheimer’s and Parkinson’s disease. Acta Neuropathol. Commun..

[67] Tavazzi E., Morrison D., Sullivan P., Morgello S., Fischer T. (2014). Brain inflammation is a common feature of HIV-infected patients without HIV encephalitis or productive brain infection. Curr. HIV Res..

[68] Gerngross L., Lehmicke G., Belkadi A., Fischer T. (2015). Role for cFMS in maintaining alternative macrophage polarization in SIV infection: Implications for HIV neuropathogenesis. J. Neuroinflammation.

[69] Walker J.A., Sulciner M.L., Nowicki K.D., Miller A.D., Burdo T.H., Williams K.C. (2014). Elevated numbers of CD163+ macrophages in hearts of simian immunodeficiency virus-infected monkeys correlate with cardiac pathology and fibrosis. AIDS Res. Hum. Retroviruses.

[70] Lee J., French B., Morgan T., French S.W. (2014). The liver is populated by a broad spectrum of markers for macrophages. In alcoholic hepatitis the macrophages are M1 and M2. Exp. Mol. Pathol..

[71] Sandahl T.D., Gronbaek H., Moller H.J., Stoy S., Thomsen K.L., Dige A.K., Agnholt J., Hamilton-Dutoit S., Thiel S., Vilstrup H. (2014). Hepatic macrophage activation and the LPS pathway in patients with alcoholic hepatitis: A prospective cohort study. Am. J. Gastroenterol..

[72] De Vito R., Alisi A., Masotti A., Ceccarelli S., Panera N., Citti A., Salata M., Valenti L., Feldstein A.E., Nobili V. (2012). Markers of activated inflammatory cells correlate with severity of liver damage in children with nonalcoholic fatty liver disease. Int. J. Mol. Med..

[73] Kazankov K., Tordjman J., Moller H.J., Vilstrup H., Poitou C., Bedossa P., Bouillot J.L., Clement K., Gronbaek H. (2015). The macrophage activation marker sCD163 is independently associated with NAFLD severity in morbid obesity and reduced by bariatric surgery. J. Gastroenterol. Hepatol..

[74] Hiraoka A., Horiike N., Akbar S.M., Michitaka K., Matsuyama T., Onji M. (2005). Expression of CD163 in the liver of patients with viral hepatitis. Pathol. Res. Pract..

[75] Baeten D., Houbiers J., Kruithof E., Vandooren B., van den Bosch F., Boots A.M., Veys E.M., Miltenburg A.M., de Keyser F. (2006). Synovial inflammation does not change in the absence of effective treatment: Implications for the use of synovial histopathology as biomarker in early phase clinical trials in rheumatoid arthritis. Ann. Rheum. Dis..

[76] Fonseca J.E., Cortez-Dias N., Francisco A., Sobral M., Canhao H., Resende C., Castelao W., Macieira C., Sequeira G., Saraiva F. (2005). Inflammatory cell infiltrate and RANKL/OPG expression in rheumatoid synovium: Comparison with other inflammatory arthropathies and correlation with outcome. Clin. Exp. Rheumatol..

[77] Baeten D., Moller H.J., Delanghe J., Veys E.M., Moestrup S.K., de Keyser F. (2004). Association of CD163+ macrophages and local production of soluble CD163 with decreased lymphocyte activation in spondylarthropathy synovitis. Arthritis Rheum..

[78] Fonseca J.E., Edwards J.C., Blades S., Goulding N.J. (2002). Macrophage subpopulations in rheumatoid synovium: Reduced CD163 expression in CD4+ T lymphocyte-rich microenvironments. Arthritis Rheum..

[79] Kjaergaard A.G., Rodgaard-Hansen S., Dige A., Krog J., Moller H.J., Tonnesen E. (2014). Monocyte expression and soluble levels of the haemoglobin receptor (CD163/sCD163) and the mannose receptor (MR/sMR) in septic and critically ill non-septic ICU patients. PLoS ONE.

[80] Vandooren B., Noordenbos T., Ambarus C., Krausz S., Cantaert T., Yeremenko N., Boumans M., Lutter R., Tak P.P., Baeten D. (2009). Absence of a classically activated macrophage cytokine signature in peripheral spondylarthritis, including psoriatic arthritis. Arthritis Rheum..

[81] Grom A.A., Mellins E.D. (2010). Macrophage activation syndrome: Advances towards understanding pathogenesis. Curr. Opin. Rheumatol..

[82] Filipovich A.H. (2009). Hemophagocytic lymphohistiocytosis (HLH) and related disorders. Hematol. Am. Soc. Hematol. Educ. Progr..

[83] Ciccia F., Alessandro R., Rizzo A., Raimondo S., Giardina A., Raiata F., Boiardi L., Cavazza A., Guggino G., de Leo G. (2013). IL-33 is overexpressed in the inflamed arteries of patients with giant cell arteritis. Ann. Rheum. Dis..

[84] Beitnes A.C., Raki M., Lundin K.E., Jahnsen J., Sollid L.M., Jahnsen F.L. (2011). Density of CD163+ CD11c+ dendritic cells increases and CD103+ dendritic cells decreases in the coeliac lesion. Scand. J. Immunol..

[85] Jiao K., Zhang J., Zhang M., Wei Y., Wu Y., Qiu Z.Y., He J., Cao Y., Hu J., Zhu H. (2013). The identification of CD163 expressing phagocytic chondrocytes in joint cartilage and its novel scavenger role in cartilage degradation. PLoS ONE.

[86] Tsuneyoshi Y., Tanaka M., Nagai T., Sunahara N., Matsuda T., Sonoda T., Ijiri K., Komiya S., Matsuyama T. (2012). Functional folate receptor beta-expressing macrophages in osteoarthritis synovium and their M1/M2 expression profiles. Scand. J. Rheumatol..

[87] Palmer M.B., Vichot A.A., Cantley L.G., Moeckel G.W. (2014). Quantification and localization of M2 macrophages in human kidneys with acute tubular injury. Int. J. Nephrol. Renov. Dis..

[88] Gutierrez E., Egido J., Rubio-Navarro A., Buendia I., Blanco Colio L.M., Toldos O., Manzarbeitia F., de Lorenzo A., Sanchez R., Ortiz A. (2012). Oxidative stress, macrophage infiltration and CD163 expression are determinants of long-term renal outcome in macrohematuria-induced acute kidney injury of IgA nephropathy. Nephron Clin. Pract..

[89] Nishiwaki S., Terakura S., Ito M., Goto T., Seto A., Watanabe K., Yanagisawa M., Imahashi N., Tsukamoto S., Shimba M. (2009). Impact of macrophage infiltration of skin lesions on survival after allogeneic stem cell transplantation: A clue to refractory graft-versus-host disease. Blood.

[90] Sekerkova A., Krepsova E., Brabcova E., Slatinska J., Viklicky O., Lanska V., Striz I. (2014). CD14+CD16+ and CD14+CD163+ monocyte subpopulations in kidney allograft transplantation. BMC Immunol..

[91] Sasaki T., Kunisaki R., Kinoshita H., Kimura H., Kodera T., Nozawa A., Hanzawa A., Shibata N., Yonezawa H., Miyajima E. (2014). Doppler ultrasound findings correlate with tissue vascularity and inflammation in surgical pathology specimens from patients with small intestinal Crohn’s disease. BMC Res. Notes.

[92] Franze E., Caruso R., Stolfi C., Sarra M., Cupi M.L., Caprioli F., Monteleone I., Zorzi F., de Nitto D., Colantoni A. (2013). Lesional accumulation of CD163-expressing cells in the gut of patients with inflammatory bowel disease. PLoS ONE.

[93] Demetter P., de Vos M., van Huysse J.A., Baeten D., Ferdinande L., Peeters H., Mielants H., Veys E.M., de Keyser F., Cuvelier C.A. (2005). Colon mucosa of patients both with spondyloarthritis and Crohn’s disease is enriched with macrophages expressing the scavenger receptor CD163. Ann. Rheum. Dis..

[94] Ratcliffe N.R., Kennedy S.M., Morganelli P.M. (2001). Immunocytochemical detection of Fcgamma receptors in human atherosclerotic lesions. Immunol. Lett..

[95] Sato T., Kameyama T., Noto T., Ueno H., Inoue H. (2015). Enhanced Expression of Hemoglobin Scavenger Receptor CD163 in Accumulated Macrophages Within Filtered Debris Between Acute Coronary Syndromes and Stable Angina Pectoris. Int. Heart J..

[96] Vogel D.Y., Vereyken E.J., Glim J.E., Heijnen P.D., Moeton M., van der Valk P., Amor S., Teunissen C.E., van Horssen J., Dijkstra C.D. (2013). Macrophages in inflammatory multiple sclerosis lesions have an intermediate activation status. J. Neuroinflammation.

[97] Zhang Z., Zhang Z.Y., Schittenhelm J., Wu Y., Meyermann R., Schluesener H.J. (2011). Parenchymal accumulation of CD163+ macrophages/microglia in multiple sclerosis brains. J. Neuroimmunol..

[98] Komohara Y., Hirahara J., Horikawa T., Kawamura K., Kiyota E., Sakashita N., Araki N., Takeya M. (2006). AM-3K, an anti-macrophage antibody, recognizes CD163, a molecule associated with an anti-inflammatory macrophage phenotype. J. Histochem. Cytochem..

[99] Kempf W., Zollinger T., Sachs M., Ullmer E., Cathomas G., Dirnhofer S., Mertz K.D. (2014). Granulomas are a source of interleukin-33 expression in pulmonary and extrapulmonary sarcoidosis. Hum. Pathol..

[100] Higashi-Kuwata N., Makino T., Inoue Y., Takeya M., Ihn H. (2009). Alternatively activated macrophages (M2 macrophages) in the skin of patient with localized scleroderma. Exp. Dermatol..

[101] Higashi-Kuwata N., Jinnin M., Makino T., Fukushima S., Inoue Y., Muchemwa F.C., Yonemura Y., Komohara Y., Takeya M., Mitsuya H. (2010). Characterization of monocyte/macrophage subsets in the skin and peripheral blood derived from patients with systemic sclerosis. Arthritis Res. Ther..

[102] Mathes A.L., Christmann R.B., Stifano G., Affandi A.J., Radstake T.R., Farina G.A., Padilla C., McLaughlin S., Lafyatis R. (2014). Global chemokine expression in systemic sclerosis (SSc): CCL19 expression correlates with vascular inflammation in SSc skin. Ann. Rheum. Dis..

[103] Kaku Y., Imaoka H., Morimatsu Y., Komohara Y., Ohnishi K., Oda H., Takenaka S., Matsuoka M., Kawayama T., Takeya M. (2014). Overexpression of CD163, CD204 and CD206 on alveolar macrophages in the lungs of patients with severe chronic obstructive pulmonary disease. PLoS ONE.

[104] Zhu H., Sun X., Zhu L., Hu F., Shi L., Li Z., Su Y. (2014). The expression and clinical significance of different forms of Mer receptor tyrosine kinase in systemic lupus erythematosus. J. Immunol. Res..

[105] Nakayama W., Jinnin M., Makino K., Kajihara I., Makino T., Fukushima S., Sakai K., Inoue Y., Ihn H. (2012). CD163 expression is increased in the involved skin and sera of patients with systemic lupus erythematosus. Eur. J. Dermatol..

[106] Suyanı E., Sucak G.T., Akyürek N., Şahin S., Baysal N.A., Yağcı M., Haznedar R. (2013). Tumor-associated macrophages as a prognostic parameter in multiple myeloma. Ann. Hematol..

[107] Niino D., Komohara Y., Murayama T., Aoki R., Kimura Y., Hashikawa K., Kiyasu J., Takeuchi M., Suefuji N., Sugita Y. (2010). Ratio of M2 macrophage expression is closely associated with poor prognosis for Angioimmunoblastic T-cell lymphoma (AITL). Pathol. Int..

[108] Sugaya M., Miyagaki T., Ohmatsu H., Suga H., Kai H., Kamata M., Fujita H., Asano Y., Tada Y., Kadono T. (2012). Association of the numbers of CD163+ cells in lesional skin and serum levels of soluble CD163 with disease progression of cutaneous T cell lymphoma. J. Dermatol. Sci..

[109] Yoon D.H., Koh Y.W., Kang H.J., Kim S., Park C.S., Lee S.W., Suh C., Huh J. (2012). CD68 and CD163 as prognostic factors for Korean patients with Hodgkin lymphoma. Eur. J. Haematol..

[110] Tan K.L., Scott D.W., Hong F., Kahl B.S., Fisher R.I., Bartlett N.L., Advani R.H., Buckstein R., Rimsza L.M., Connors J.M. (2012). Tumor-associated macrophages predict inferior outcomes in classic Hodgkin lymphoma: A correlative study from the E2496 Intergroup trial. Blood.

[111] Zaki M.A., Wada N., Ikeda J., Shibayama H., Hashimoto K., Yamagami T., Tatsumi Y., Tsukaguchi M., Take H., Tsudo M. (2011). Prognostic implication of types of tumor-associated macrophages in Hodgkin lymphoma. Virchows Arch..

[112] Clear A.J., Lee A.M., Calaminici M., Ramsay A.G., Morris K.J., Hallam S., Kelly G., MacDougall F., Lister T.A., Gribben J.G. (2010). Increased angiogenic sprouting in poor prognosis FL is associated with elevated numbers of CD163+ macrophages within the immediate sprouting microenvironment. Blood.

[113] Kanno H., Nishihara H., Wang L., Yuzawa S., Kobayashi H., Tsuda M., Kimura T., Tanino M., Terasaka S., Tanaka S. (2013). Expression of CD163 prevents apoptosis through the production of granulocyte colony-stimulating factor in meningioma. Neuro-Oncology.

[114] Grund S., Schittenhelm J., Roser F., Tatagiba M., Mawrin C., Kim Y., Bornemann A. (2009). The microglial/macrophagic response at the tumour–brain border of invasive meningiomas. Neuropathol. Appl. Neurobiol..

[115] Prosniak M., Harshyne L.A., Andrews D.W., Kenyon L.C., Bedelbaeva K., Apanasovich T.V., Heber-Katz E., Curtis M.T., Cotzia P., Hooper D.C. (2013). Glioma grade is associated with the accumulation and activity of cells bearing M2 monocyte markers. Clin. Cancer Res..

[116] Lan C., Huang X., Lin S., Huang H., Cai Q., Wan T., Lu J., Liu J. (2013). Expression of M2-polarized macrophages is associated with poor prognosis for advanced epithelial ovarian cancer. Technol. Cancer Res. Treat..

[117] Ohri C.M., Shikotra A., Green R.H., Waller D.A., Bradding P. (2011). The tissue microlocalisation and cellular expression of CD163, VEGF, HLA-DR, iNOS, and MRP 8/14 is correlated to clinical outcome in NSCLC. PLoS ONE.

[118] Kurahara H., Shinchi H., Mataki Y., Maemura K., Noma H., Kubo F., Sakoda M., Ueno S., Natsugoe S., Takao S. (2011). Significance of M2-polarized tumor-associated macrophage in pancreatic cancer. J. Surg. Res..

[119] Medrek C., Ponten F., Jirstrom K., Leandersson K. (2012). The presence of tumor associated macrophages in tumor stroma as a prognostic marker for breast cancer patients. BMC Cancer.

[120] Tang X. (2013). Tumor-associated macrophages as potential diagnostic and prognostic biomarkers in breast cancer. Cancer Lett..

[121] Lima L., Oliveira D., Tavares A., Amaro T., Cruz R., Oliveira M.J., Ferreira J.A., Santos L. (2014). The predominance of M2-polarized macrophages in the stroma of low-hypoxic bladder tumors is associated with BCG immunotherapy failure. Urol. Oncol..

[122] He K.F., Zhang L., Huang C.F., Ma S.R., Wang Y.F., Wang W.M., Zhao Z.L., Liu B., Zhao Y.F., Zhang W.F. (2014). CD163+ tumor-associated macrophages correlated with poor prognosis and cancer stem cells in oral squamous cell carcinoma. Biomed. Res. Int..

[123] Fujii N., Shomori K., Shiomi T., Nakabayashi M., Takeda C., Ryoke K., Ito H. (2012). Cancer-associated fibroblasts and CD163-positive macrophages in oral squamous cell carcinoma: Their clinicopathological and prognostic significance. J. Oral Pathol. Med..

[124] Shabo I., Olsson H., Elkarim R., Sun X.F., Svanvik J. (2014). Macrophage Infiltration in Tumor Stroma is Related to Tumor Cell Expression of CD163 in Colorectal Cancer. Cancer Microenviron..

[125] Behnes C.L., Bremmer F., Hemmerlein B., Strauss A., Strobel P., Radzun H.J. (2014). Tumor-associated macrophages are involved in tumor progression in papillary renal cell carcinoma. Virchows Arch..

[126] Komohara Y., Hasita H., Ohnishi K., Fujiwara Y., Suzu S., Eto M., Takeya M. (2011). Macrophage infiltration and its prognostic relevance in clear cell renal cell carcinoma. Cancer Sci..

[127] Kubler K., Ayub T.H., Weber S.K., Zivanovic O., Abramian A., Keyver-Paik M.D., Mallmann M.R., Kaiser C., Serce N.B., Kuhn W. (2014). Prognostic significance of tumor-associated macrophages in endometrial adenocarcinoma. Gynecol. Oncol..

[128] Hasita H., Komohara Y., Okabe H., Masuda T., Ohnishi K., Lei X.F., Beppu T., Baba H., Takeya M. (2010). Significance of alternatively activated macrophages in patients with intrahepatic cholangiocarcinoma. Cancer Sci..

[129] Bronkhorst I.H., Ly L.V., Jordanova E.S., Vrolijk J., Versluis M., Luyten G.P., Jager M.J. (2011). Detection of M2-macrophages in uveal melanoma and relation with survival. Investig. Ophthalmol. Vis. Sci..

[130] Emri E., Egervari K., Varvolgyi T., Rozsa D., Miko E., Dezso B., Veres I., Mehes G., Emri G., Remenyik E. (2013). Correlation among metallothionein expression, intratumoural macrophage infiltration and the risk of metastasis in human cutaneous malignant melanoma. J. Eur. Acad. Dermatol. Venereol..

[131] Jensen T.O., Schmidt H., Moller H.J., Hoyer M., Maniecki M.B., Sjoegren P., Christensen I.J., Steiniche T. (2009). Macrophage markers in serum and tumor have prognostic impact in American Joint Committee on Cancer stage I/II melanoma. J. Clin. Oncol..

[132] Shabo I., Svanvik J. (2011). Expression of macrophage antigens by tumor cells. Adv. Exp. Med. Biol..

[133] Larizza L., Schirrmacher V., Pfluger E. (1984). Acquisition of high metastatic capacity after *in vitro* fusion of a nonmetastatic tumor line with a bone marrow-derived macrophage. J. Exp. Med..

[134] Munzarova M., Lauerova L., Capkova J. (1992). Are advanced malignant melanoma cells hybrids between melanocytes and macrophages?. Melanoma Res..

[135] Maniecki M.B., Etzerodt A., Ulhoi B.P., Steiniche T., Borre M., Dyrskjot L., Orntoft T.F., Moestrup S.K., Moller H.J. (2012). Tumor-promoting macrophages induce the expression of the macrophage-specific receptor CD163 in malignant cells. Int. J. Cancer.

[136] Chakraborty A.K., de Freitas Sousa J., Espreafico E.M., Pawelek J.M. (2001). Human monocyte x mouse melanoma fusion hybrids express human gene. Gene.

[137] Huysentruyt L.C., Mukherjee P., Banerjee D., Shelton L.M., Seyfried T.N. (2008). Metastatic cancer cells with macrophage properties: Evidence from a new murine tumor model. Int. J. Cancer.

[138] Busund L.T., Killie M.K., Bartnes K., Seljelid R. (2002). Spontaneously formed tumorigenic hybrids of Meth A sarcoma and macrophages grow faster and are better vascularized than the parental tumor. Int. J. Cancer.

[139] Pawelek J.M. (2000). Tumour cell hybridization and metastasis revisited. Melanoma Res..

[140] Chakraborty A.K., Pawelek J., Ikeda Y., Miyoshi E., Kolesnikova N., Funasaka Y., Ichihashi M., Taniguchi N. (2001). Fusion hybrids with macrophage and melanoma cells up-regulate N-acetylglucosaminyltransferase V, beta1-6 branching, and metastasis. Cell Growth Differ..

[141] Shabo I., Stal O., Olsson H., Dore S., Svanvik J. (2008). Breast cancer expression of CD163, a macrophage scavenger receptor, is related to early distant recurrence and reduced patient survival. Int. J. Cancer.

[142] Shabo I., Olsson H., Sun X.F., Svanvik J. (2009). Expression of the macrophage antigen CD163 in rectal cancer cells is associated with early local recurrence and reduced survival time. Int. J. Cancer.

[143] Pancione M., Giordano G., Remo A., Febbraro A., Sabatino L., Manfrin E., Ceccarelli M., Colantuoni V. (2014). Immune escape mechanisms in colorectal cancer pathogenesis and liver metastasis. J. Immunol. Res..

[144] Yoshida C., Takeuchi M. (2008). Histiocytic sarcoma: Identification of its histiocytic origin using immunohistochemistry. Intern. Med..

[145] Garcia C., Gardner D., Reichard K.K. (2008). CD163: A specific immunohistochemical marker for acute myeloid leukemia with monocytic differentiation. Appl. Immunohistochem. Mol. Morphol..

[146] Bachli E.B., Schaer D.J., Walter R.B., Fehr J., Schoedon G. (2006). Functional expression of the CD163 scavenger receptor on acute myeloid leukemia cells of monocytic lineage. J. Leukoc. Biol..

[147] Adair J.R., Howard P.W., Hartley J.A., Williams D.G., Chester K.A. (2012). Antibody-drug conjugates-a perfect synergy. Expert Opin. Biol. Ther..

[148] Harper J., Mao S., Strout P., Kamal A. (2013). Selecting an optimal antibody for antibody-drug conjugate therapy: Internalization and intracellular localization. Methods Mol. Biol..

[149] Granfeldt A., Hvas C.L., Graversen J.H., Christensen P.A., Petersen M.D., Anton G., Svendsen P., Solling C., Etzerodt A., Tonnesen E. (2013). Targeting dexamethasone to macrophages in a porcine endotoxemic model. Crit. Care Med..

[150] Graversen J.H., Svendsen P., Dagnaes-Hansen F., Dal J., Anton G., Etzerodt A., Petersen M.D., Christensen P.A., Moller H.J., Moestrup S.K. (2012). Targeting the hemoglobin scavenger receptor CD163 in macrophages highly increases the anti-inflammatory potency of dexamethasone. Mol. Ther..

[151] Eichendorff S., Svendsen P., Bender D., Keiding S., Christensen E.I., Deleuran B., Moestrup S.K. (2015). Biodistribution and PET imaging of a novel [68Ga]-anti-CD163-antibody conjugate in rats with collagen-induced arthritis and in controls. Mol. Imaging Biol..

[152] Allen T.M., Hansen C. (1991). Pharmacokinetics of stealth *versus* conventional liposomes: Effect of dose. Biochim. Biophys. Acta.

[153] Torchilin V.P., Levchenko T.S., Lukyanov A.N., Khaw B.A., Klibanov A.L., Rammohan R., Samokhin G.P., Whiteman K.R. (2001). p-Nitrophenylcarbonyl-PEG-PE-liposomes: Fast and simple attachment of specific ligands, including monoclonal antibodies, to distal ends of PEG chains via p-nitrophenylcarbonyl groups. Biochim. Biophys. Acta.

[154] Etzerodt A., Maniecki M.B., Graversen J.H., Moller H.J., Torchilin V.P., Moestrup S.K. (2012). Efficient intracellular drug-targeting of macrophages using stealth liposomes directed to the hemoglobin scavenger receptor CD163. J. Control. Release.

[155] Zhang N., Palmer A.F. (2012). Liposomes surface conjugated with human hemoglobin target delivery to macrophages. Biotechnol. Bioeng..

[156] Earp J.C., DuBois D.C., Molano D.S., Pyszczynski N.A., Almon R.R., Jusko W.J. (2008). Modeling Corticosteroid Effects in a Rat Model of Rheumatoid Arthritis II: Mechanistic Pharmacodynamic Model for Dexamethasone Effects in Lewis Rats with Collagen-Induced Arthritis. J. Pharmacol. Exp. Ther..

[157] Samtani M.N., Jusko W.J. (2007). Quantification of dexamethasone and corticosterone in rat biofluids and fetal tissue using highly sensitive analytical methods: Assay validation and application to a pharmacokinetic study. Biomed. Chromatogr..

[158] Bartlett D.W., Davis M.E. (2006). Insights into the kinetics of siRNA-mediated gene silencing from live-cell and live-animal bioluminescent imaging. Nucleic Acids Res..

[159] Falchi M., Varricchio L., Martelli F., Masiello F., Federici G., Zingariello M., Girelli G., Whitsett C., Petricoin E.F., Moestrup S.K. (2015). Dexamethasone targeted directly to macrophages induces macrophage niches that promote erythroid expansion. Haematologica.

[160] Fritz J.M., Tennis M.A., Orlicky D.J., Lin H., Ju C., Redente E.F., Choo K.S., Staab T.A., Bouchard R.J., Merrick D.T. (2014). Depletion of tumor-associated macrophages slows the growth of chemically induced mouse lung adenocarcinomas. Front. Immunol..

[161] Wu X., Schulte B.C., Zhou Y., Haribhai D., Mackinnon A.C., Plaza J.A., Williams C.B., Hwang S.T. (2014). Depletion of M2-like tumor-associated macrophages delays cutaneous T-cell lymphoma development *in vivo*. J. Investig. Dermatol..

[162] Reusser N.M., Dalton H.J., Pradeep S., Gonzalez-Villasana V., Jennings N.B., Vasquez H.G., Wen Y., Rupaimoole R., Nagaraja A.S., Gharpure K. (2014). Clodronate inhibits tumor angiogenesis in mouse models of ovarian cancer. Cancer Biol. Ther..

[163] Zeisberger S.M., Odermatt B., Marty C., Zehnder-Fjallman A.H., Ballmer-Hofer K., Schwendener R.A. (2006). Clodronate-liposome-mediated depletion of tumour-associated macrophages: A new and highly effective antiangiogenic therapy approach. Br. J. Cancer.

[164] Mathes M., Jordan M., Dow S. (2006). Evaluation of liposomal clodronate in experimental spontaneous autoimmune hemolytic anemia in dogs. Exp. Hematol..

[165] Guth A.M., Hafeman S.D., Elmslie R.E., Dow S.W. (2013). Liposomal clodronate treatment for tumour macrophage depletion in dogs with soft-tissue sarcoma. Vet. Comp. Oncol..

[166] Hafeman S., London C., Elmslie R., Dow S. (2010). Evaluation of liposomal clodronate for treatment of malignant histiocytosis in dogs. Cancer Immunol. Immunother..

[167] Kim H.M., Lee Y.W., Lee K.J., Kim H.S., Cho S.W., van Rooijen N., Guan Y., Seo S.H. (2008). Alveolar macrophages are indispensable for controlling influenza viruses in lungs of pigs. J. Virol..

[168] Reed J.L., Brewah Y.A., Delaney T., Welliver T., Burwell T., Benjamin E., Kuta E., Kozhich A., McKinney L., Suzich J. (2008). Macrophage impairment underlies airway occlusion in primary respiratory syncytial virus bronchiolitis. J. Infect. Dis..

[169] Moghimi S.M., Parhamifar L., Ahmadvand D., Wibroe P.P., Andresen T.L., Farhangrazi Z.S., Hunter A.C. (2012). Particulate systems for targeting of macrophages: Basic and therapeutic concepts. J. Innate Immun..

[170] Kim S.S., Ye C., Kumar P., Chiu I., Subramanya S., Wu H., Shankar P., Manjunath N. (2010). Targeted delivery of siRNA to macrophages for anti-inflammatory treatment. Mol. Ther..

[171] Ye C., Bhan A.K., Deshpande V., Shankar P., Manjunath N. (2013). Silencing TNF-alpha in macrophages and dendritic cells for arthritis treatment. Scand. J. Rheumatol..

[172] Mochizuki S., Sakurai K. (2011). Dectin-1 targeting delivery of TNF-alpha antisense ODNs complexed with beta-1,3-glucan protects mice from LPS-induced hepatitis. J. Control. Release.

[173] Melgert B.N., Olinga P., van der Laan J.M., Weert B., Cho J., Schuppan D., Groothuis G.M., Meijer D.K., Poelstra K. (2001). Targeting dexamethasone to Kupffer cells: Effects on liver inflammation and fibrosis in rats. Hepatology.

[174] Wijagkanalan W., Kawakami S., Takenaga M., Igarashi R., Yamashita F., Hashida M. (2008). Efficient targeting to alveolar macrophages by intratracheal administration of mannosylated liposomes in rats. J. Control. Release.

[175] Wijagkanalan W., Higuchi Y., Kawakami S., Teshima M., Sasaki H., Hashida M. (2008). Enhanced anti-inflammation of inhaled dexamethasone palmitate using mannosylated liposomes in an endotoxin-induced lung inflammation model. Mol. Pharmacol..

[176] Martinez-Pomares L. (2012). The mannose receptor. J. Leukoc. Biol..

[177] Ozbakir B., Crielaard B.J., Metselaar J.M., Storm G., Lammers T. (2014). Liposomal corticosteroids for the treatment of inflammatory disorders and cancer. J. Control. Release.

[178] Hofkens W., Grevers L.C., Walgreen B., de Vries T.J., Leenen P.J., Everts V., Storm G., van den Berg W.B., van Lent P.L. (2011). Intravenously delivered glucocorticoid liposomes inhibit osteoclast activity and bone erosion in murine antigen-induced arthritis. J. Control. Release.

[179] Avnir Y., Turjeman K., Tulchinsky D., Sigal A., Kizelsztein P., Tzemach D., Gabizon A., Barenholz Y. (2011). Fabrication principles and their contribution to the superior *in vivo* therapeutic efficacy of nano-liposomes remote loaded with glucocorticoids. PLoS ONE.

[180] Avnir Y., Ulmansky R., Wasserman V., Even-Chen S., Broyer M., Barenholz Y., Naparstek Y. (2008). Amphipathic weak acid glucocorticoid prodrugs remote-loaded into sterically stabilized nanoliposomes evaluated in arthritic rats and in a Beagle dog: A novel approach to treating autoimmune arthritis. Arthritis Rheum..

[181] Ulmansky R., Turjeman K., Baru M., Katzavian G., Harel M., Sigal A., Naparstek Y., Barenholz Y. (2012). Glucocorticoids in nano-liposomes administered intravenously and subcutaneously to adjuvant arthritis rats are superior to the free drugs in suppressing arthritis and inflammatory cytokines. J. Control. Release.

[182] Bartneck M., Scheyda K.M., Warzecha K.T., Rizzo L.Y., Hittatiya K., Luedde T., Storm G., Trautwein C., Lammers T., Tacke F. (2015). Fluorescent cell-traceable dexamethasone-loaded liposomes for the treatment of inflammatory liver diseases. Biomaterials.

[183] Seki E., Schnabl B. (2012). Role of innate immunity and the microbiota in liver fibrosis: Crosstalk between the liver and gut. J. Physiol..

[184] Gao B., Bataller R. (2011). Alcoholic liver disease: Pathogenesis and new therapeutic targets. Gastroenterology.

[185] Cantini F., Nannini C., Niccoli L., Iannone F., Delogu G., Garlaschi G., Sanduzzi A., Matucci A., Prignano F., Conversano M. (2015). Guidance for the management of patients with latent tuberculosis infection requiring biologic therapy in rheumatology and dermatology clinical practice. Autoimmun. Rev..

